# Targeting the Regulatory Subunit R2Alpha of Protein Kinase A in Human Glioblastoma through shRNA-Expressing Lentiviral Vectors

**DOI:** 10.3390/v13071361

**Published:** 2021-07-13

**Authors:** Maira Zorzan, Claudia Del Vecchio, Stefania Vogiatzis, Elisa Saccon, Cristina Parolin, Giorgio Palù, Arianna Calistri, Carla Mucignat-Caretta

**Affiliations:** Department of Molecular Medicine, University of Padova, Via Gabelli 63, 35121 Padova, Italy; mairazorzan@gmail.com (M.Z.); claudia.delvecchio@unipd.it (C.D.V.); stefi.voy@virgilio.it (S.V.); elisa.saccon.1@phd.unipd.it (E.S.); cristina.parolin@unipd.it (C.P.); giorgio.palu@unipd.it (G.P.)

**Keywords:** lentiviral vectors, glioblastoma, protein kinase A, brain tumor, short hairpin RNA, cAMP

## Abstract

Glioblastoma is the most malignant and most common form of brain tumor, still today associated with a poor 14-months median survival from diagnosis. Protein kinase A, particularly its regulatory subunit R2Alpha, presents a typical intracellular distribution in glioblastoma cells compared to the healthy brain parenchyma and this peculiarity might be exploited in a therapeutic setting. In the present study, a third-generation lentiviral system for delivery of shRNA targeting the regulatory subunit R2Alpha of protein kinase A was developed. Generated lentiviral vectors are able to induce an efficient and stable downregulation of R2Alpha in different cellular models, including non-stem and stem-like glioblastoma cells. In addition, our data suggest a potential correlation between silencing of the regulatory subunit of protein kinase A and reduced viability of tumor cells, apparently due to a reduction in replication rate. Thus, our findings support the role of protein kinase A as a promising target for novel anti-glioma therapies.

## 1. Introduction

Glioblastoma (GBM) is the most malignant and most common form of glioma within the brain, with an incidence of around 12,000 cases every year in the United States [1]. Despite very aggressive treatment including surgery, radiotherapy, and chemotherapy [2,3,4], this brain tumor is associated with a modest 27% survival two years after diagnosis [5], the overall median survival for GBM patients being still as poor as 14 months from diagnosis [6]. The remarkable intratumor heterogeneity, the high infiltrative nature, and the anatomical location of GBM strongly limit the efficacy of conventional anti-tumor therapies [7,8] and pose severe obstacles to the success of even the most recent therapeutic strategies. The presence of radio- and chemo-resistant cancer stem cells and their known plasticity further contribute to the striking failure of current glioblastoma treatments [9,10,11,12,13,14,15] and new therapeutic options are urgently required. For these reasons, pioneering therapeutic strategies have been designed, including the improved delivery of small drugs and monoclonal antibodies, RNA-based strategies, immunotherapies, and viral gene therapies [16,17,18]. On the other hand, new molecular targets have been identified among the components of the signaling pathways specifically altered in brain cancers, such as IDH, PTEN, PI3K/Akt/mTOR, EGF, Notch, VEGF, PDGF, SHH, and TGF-β [19]. In this scenario, protein kinase A (PKA), which presents relevant peculiarities in GBM, has emerged as a promising target for novel anti-cancer and anti-glioma therapies [20,21]. Particularly, the regulatory subunit R2Alpha (R2A) of PKA was previously demonstrated to be specifically present in perinuclear clusters co-localized with the Golgi apparatus in rodent and human GBM cells, whereas this peculiar distribution was not detected in the healthy brain parenchyma [22,23]. Upregulation of R2A expression in GBM specimens compared to non-tumor brain tissue and poor survival of patients showing the highest R2A expression have also been reported [22]. The functional significance of Golgi-associated PKA in GBM cells and other cancer and non-cancer cell lines is still unclear [24] and needs further investigation to reveal potential applications in cancer treatment. In order to give additional insights into the role of PKA R2A subunit in human GBM, we developed a third-generation lentiviral system for delivery of short hairpin RNA (shRNA) targeting PKA R2A in human GBM cellular models. 

## 2. Materials and Methods

### 2.1. Cell Lines and Cell Cultures 

Human embryonic kidney 293T (ATCC CRL-3216) and the glioblastoma U87MG (herein named U87, ATCC HTB-14) cell lines were grown in Dulbecco’s modified Eagle’s medium (Thermo Fisher Scientific, Rodano, Italy) supplemented with 10% heat-inactivated fetal bovine serum, FBS (Thermo Fisher Scientific). Adrenocortical carcinoma SW13 cells (ATCC CCL-105) were cultivated in Leibovitz’s L-15 Medium, 10% FBS. The human glioblastoma (GBM) stem cell like culture NCH421K (CLS Cell Lines Service GmbH) [25] was also employed. NCH421K derives from a glioblastoma of a 66-year-old man, tumorigenic and highly CD133 positive. Primary cells were obtained from surgical specimen of a glioblastoma patient (Caucasian, female, age 63), experiment was conducted according to the Helsinki declaration and its amendments. Ethical Committee approval from the Comitato Etico per la Sperimentazione Clinica (protocol 1883P) and informed consent from the patient were acquired. GBM stem cell like cultures were grown as cell clusters (spheres) in suspension in Neurobasal medium (Thermo Fisher Scientific) supplemented with 3 mmoL/L L-Glutamine (Sigma-Aldrich, Milan, Italy), 1X B27 supplement (Thermo Fisher Scientific), 0.5X N2 supplement (Thermo Fisher Scientific), 2 μg/mL heparin (STEMCELL Technologies Cologne, Germany), 20 ng/mL recombinant human EGF (Merck Millipore, Milan, Italy), 20 ng/mL recombinant human FGF-basic (Peprotech, Hamburg, Germany), 1% Penicillin/Streptomycin (Thermo Fisher Scientific), 1% *v*/*v* Tetracycline (Cell Biolabs, Segrate, Italy), and 1% *v*/*v* Plasmocin (InVivogen, Rome, Italy). 

### 2.2. Construction of Self-Inactivating Lentiviral Vectors Expressing PKA R2A Specific shRNAs

The sequence of 97-mer oligonucleotide (5’-3’) was for PKA R2A Temp1: TGCTGTTGACAGTGAGCGCAGCAGATTTAATAGACGAGTATAGTGAAGCCACAGATGTA**TACTCGTCTATTAAATCTGCTA**TGCCTACTGCCTCGGA, and for Temp2: TGCTGTTGACAGTGAGCGACCGCTCTGTTGGTCAATATGATAGTGAAGCCACAGATGTA**TCATATTGACCAACAGAGCGGG**TGCCTACTGCCTCGGA (guide in bold and passenger strands underlined). PKA R2A Temp1 and Temp2 shRNA were PCR amplified using the primers miRE-Xho-fw (TGAACTCGAGAAGGTATATTGCTGTTGACAGTGAGCG) and miRE-EcoOligo-rev (TCTCGAATTCTAGCCCCTTGAAGTCCGAGGCAGTAGGC), 0.05 ng oligonucleotide template and the PfuUltra™ HF DNA polymerase (Agilent Technologies, Cernusco sul Naviglio, Italy). A scrambled control shRNA was also designed, by means of the GenScript program (https://www.genscript.com/ssl-bin/app/scramble, accessed on 25 March 2017) TGCTGTTGACAGTGAGCGCTAATTAACGAGACAGGTTAATTAGTGAAGCCACAGATGTA**ATTAACCTGTCTCGTTAATTAC**TGCCTACTGCCTCGGA (guide and passenger strands in bold and underlined, respectively). The absence of cellular targets was further verified by use of the SpliceCenter/siRNACheck software (http://projects.insilico.us/SpliceCenter/siRNACheck.jsp, accessed on 25 March 2017), which confirmed that no human gene splice variants were targeted by the designed scrambled sequence. Following amplification, the PCR fragments were XhoI/EcoRI cloned into the eukaryotic expression plasmid pEGFP-C1 (Addgene, Watertown, MA, USA). The third-generation pRRLsin18.cPPT.hCMV.GFP.Wpre self-inactivating lentiviral vector (hereafter named cPPT.hCMV.GFP), which contains a green fluorescent protein (GFP) reporter gene, was kindly provided by L. Naldini and was used to develop the anti-PKA R2A constructs. In particular, two parallel cloning strategies were adopted to generate recombinant lentiviral vectors for expression of either transcripts encoding for Temp1 and Temp2 shRNAs or polycistronic sequences expressing both GFP and PKA R2A shRNAs. For generating the cPPT.hCMV.shTemp1 and cPPT.hCMV.shTemp2 vectors, recombinant pEGFP-C1-shTemp1 and pEGFP-C1-shTemp2 were submitted to BglII/SalI restriction reactions and shRNA fragments Temp1 and Temp2 were cloned into BamHI/SalI digested cPPT.hCMV.GFP vector. The obtained constructs express PKA R2A shRNA Temp1 or Temp2 under the control of hCMV promoter and do not express the green fluorescent protein. For the development of cPPT.hCMV.EGFP.shTemp1 and cPPT.hCMV.EGFP.shTemp2 vectors, the pEGFP-C1-shTemp1 and pEGFP-C1-shTemp2 plasmids were submitted to NheI restriction, blunting of overhangs, and SalI restriction to generate fragments including the enhanced (E)GFP gene and the Temp1 or Temp2 shRNA sequence. These fragments were then cloned into the cPPT.hCMV.GFP vector previously submitted to BamHI restriction, blunting of overhangs, and SalI restriction. For generating lentiviral vectors expressing the designed scrambled shRNA, after amplification, the PCR fragment was cloned in the pCR2.1-TOPO vector (Thermo Fisher Scientific), following the manufacturers’ instructions. The scrambled shRNA insert from selected recombinant pCR2.1-TOPO vectors was then XhoI/EcoRI cloned into the eukaryotic expression plasmid pEGFP-C1, generating the pEGFP-C1-shSCR. Similar to previous cloning of PKA R2A shRNAs, recombinant pEGF-C1-shSCR was submitted to BglII/SalI restriction reaction and shSCR fragment was cloned into BamHI/SalI digested cPPT.hCMV.GFP vector. The obtained cPPT.hCMV.shSCR vector expresses the designed scrambled shRNA under the control of hCMV promoter and does not express the green fluorescent protein as the GFP gene was deleted by BamHI/SalI restriction of the lentiviral vector. All constructs were verified by restriction analysis and sequencing.

### 2.3. Transfection of 293T Cells with Lentiviral Vectors Expressing PKA R2A Specific shRNA, Recombinant Lentiviral Particle Production and Transduction of Target Cells

For transfection of 293T cells with PKA R2A gene silencing lentiviral vectors, 500 ng of pRSV-Rev plasmid were used together with 1500 ng of lentiviral transfer vector, by Lipofectamine 2000 (Thermo Fisher Scientific), following manufacturers’ instructions. Of note, cell medium containing transfection reagents was replaced with DMEM 10% FBS 4 h after transfection. 

Vesicular stomatitis virus (VSV)-G pseudotyped vector stocks were produced by calcium phosphate transfection of 293T cells, as previously reported [26]. Briefly, 24 h before transfection, 12 × 10^6^ 293T cells were seeded in T-150 culture flasks and then cotransfected with 20 µg of the appropriate gene transfer vector, 10 µg of pMDLg/pRRE #54, 10 µg of pRSV-REV and 10 µg of pMD2.VSV.G. All plasmids were kindly provided by L. Naldini. The culture supernatants were collected on day 2 post-transfection, filtered with a 0.45-µm-pore-size membrane and stored at −80 °C until use. When required, vector particles in the supernatants were concentrated by ultracentrifugation (27,000 rpm, 2 h, 4 °C). Reverse transcriptase (RT) activity was determined as previously described [27]. 

Target cells were transduced as follows: adherent cells (10^5^ U87 cells/well or 2.5 × 10^5^ 293 T cells/well in 6-well plates) were grown for 24 h and then incubated with 1 mL of lentiviral vector-containing medium or proper dilutions of concentrated lentiviral preparations. One additional mL of DMEM 10% FBS was added 8 h later and cells were evaluated at least 72 h post-transduction. 

To evaluate cell proliferation, U87 cells were seeded (0.5 × 10^6^ /well in 6-well plates) and 24 h later transduced with shTemp1, shTemp2, and shSCR recombinant lentiviral particles (200,000 cpm). After 11 and 13 days, 2 or 5 × 10^5^ cells were seeded in 96-well plates, after 4 h the medium was removed and cells were analyzed by the CyQUANT NF Cell Proliferation Assay, (Thermo Fisher Scientific), following the Manufacturers’ instructions. Values were normalized to the ones obtained for cells transduced with the scrambled vector.

To evaluate cell death, 0.5 × 10^6^ U87 cells were seeded in 6-well plates and 24 h later transduced with shTemp1, shTemp2, and shSCR recombinant lentiviral particles (200,000 cpm). Thirteen days after, cells were analyzed with FACS using propidium iodide/Annexin V Apoptosis Detection Kit FITC (Thermo Fisher Scientific).

For human GBM spheres, proper dilutions of concentrated lentiviral vectors in complete Neurobasal medium were incubated with GBM cells immediately after mechanical dissociation of the spheres. In all experiments mock transduction was performed with DMEM 10% FBS or complete Neurobasal medium, for 293T and U87 or NCH421K cell experiments, respectively. The amount of recombinant lentiviral particles employed in each experiment is reported in the text. All transduced cells were routinely observed under a direct light or fluorescent inverted microscope (Leica DMIL LED) to evaluate eventual effects on cell morphology and proliferation (by cell counting after trypan blue staining) and to monitor EGFP expression, where possible.

### 2.4. MTT Assay 

MTT assay was performed on U87 cells transduced with PKA R2A and scrambled shRNA lentiviral vectors using Cell Proliferation Kit I (MTT) (Roche Italia, Monza, Italy) according to manufacturer’s instructions. Briefly, U87 cells (10^3^ cells/well in 96-well plates) were transduced as above; on day 11 post-transduction they were processed for Western blot analysis of PKA R2A expression and seeded (10^3^ cells/well in 96-well plates) for MTT assays. On day 12, 14, 16, and 18 post-transduction, 10 μL of the MTT labeling reagent were added to each well and cells were returned to incubator. After 4 h incubation, 100 μL of the solubilization solution was added into each well. The well plate was allowed to stand overnight in the incubator for complete solubilization of formazan crystals and the absorbance of the samples at 620 nm was measured with a Tecan Sunrise absorbance microplate reader. 

### 2.5. Cell Lysates and Western Blot Analysis

Cell lysates of lentiviral transduced cells were prepared and quantified for subsequent analysis on Sodium Dodecyl Sulphate—PolyAcrylamide Gel Electrophoresis (SDS-PAGE) and Western blot. At defined time points transduced cells were lysed in RIPA buffer (10% *v*/*v* PBS 10X, 1% *v*/*v* IGEPAL CA-630, 0.5% *v*/*v* sodium deoxycholate, 0.05% *v*/*v* SDS in milliQ water) supplemented with protease inhibitor cocktail (cOmplete™ Protease Inhibitor Cocktail, Merck Sigma-Aldrich). Quantification of protein extracts was performed through a bicinchoninic acid (BCA) protein assay (Micro BCA Protein Assay Kit, Thermo Fisher Scientific), following manufacturers’ instructions. Fifteen to forty micrograms of protein extracts were subjected to SDS-PAGE, followed by Western blot analysis. Incubation of the nitrocellulose membrane with the primary antibodies, properly diluted in blocking solution (5% *w*/*v* dry milk (Blotting-Grade Blocker, Bio-Rad) in PBS + 0.1% *v*/*v* Tween 20), was performed on shaking overnight at 4 °C, while incubation with secondary antibodies was done 1 h at room temperature. Proteins were revealed through chemiluminescence using ECL reagents (Amersham ECL Western Blotting Detection Reagents, Merck Sigma Aldrich) according to manufacturer’s instructions. The following primary antibodies were used: PKA R2A, R1A, Catalytic alpha subunit (all three made in rabbit, 1:1000; Santa Cruz Biotechnology, Segrate, Italy) and GAPDH (rabbit, 1:7500; Merck Sigma-Aldrich). The anti-rabbit peroxidase-conjugated (goat, 1:1000; Abcam, Cambridge, UK) secondary antibody was used for chemiluminescent detection. Representative Western blots are shown in the figures. Quantification of Western blot was done with ImageJ software (Analyze Gels tool) to extract the relative density of bands. Each PKA R2A band was expressed as a percentage of GAPDH in the same lane; the reduction in expression at different time points was calculated as a percentage relative to the control conditions at the same time point, as specified in the figures. 

### 2.6. Gene Expression Data

The Cancer Genome Atlas (TCGA) glioblastoma database was interrogated with UCSC Xena browser [28]. Gene expression was determined using the Illumina HiSeq2000 RNA Sequencing platform on 172 samples, details are available online: https://xenabrowser.net/datapages/?dataset=TCGA.GBM.sampleMap%2FHiSeqV2&host=https%3A%2F%2Ftcga.xenahubs.net&removeHub=https%3A%2F%2Fxena.treehouse.gi.ucsc.edu%3A443, accessed on 24 May 2021.

### 2.7. Statistical Analysis

Statistical analysis of data was performed with the software GraphPad Prism 5 (GraphPad Software Inc., San Diego, CA, USA).

## 3. Results

**Figure 1:**
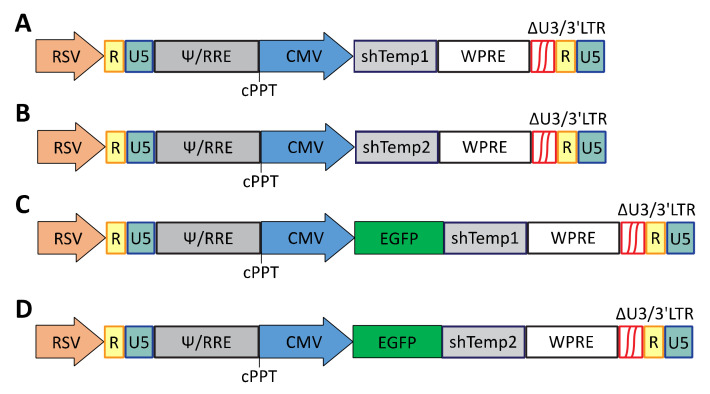
Schematic representation of the cPPT.hCMV.GFP-based lentiviral vectors for silencing of PKA R2A expression. (**A**) cPPT.hCMV.shTemp1; (**B**) cPPT.hCMV.shTemp2; (**C**) cPPT.hCMV.EGFP.shTemp1; (**D**) cPPT.hCMV.EGFP.shTemp2.

Fellmann and colleagues identified an optimized miRNA backbone, termed “miRE”, which strongly increases knockdown efficacy through enhanced pri-miRNA processing and consequent higher mature small RNA levels [29]. For different human and mouse coding genes they also provided a list of ten 97-mer oligonucleotides for shRNA predicted to target all known transcript variants [29]. In the present study, top-ranked oligonucleotides Temp1 and Temp2 for PKA R2A shRNA were selected for generation of recombinant gene-silencing lentiviral vectors. Specifically, the cPPT.hCMV.GFP lentiviral vector was engineered for expression of either PKA R2A Temp1 (shTemp1) or PKA R2A Temp2 (shTemp2) shRNA, as well as for the co-expression of either shTemp1 or shTemp2 with EGFP reporter protein. In the latter case, the generated recombinant constructs will express polycistronic transcripts encoding both for the EGFP and the PKA R2A shRNA Temp1 or Temp2 [29]. Oligonucleotide sequences for shTemp1 and shTemp2 shRNAs were predicted to target all known transcript variants of human PKA R2A gene [30]. A schematic representation of the generated recombinant vectors expressing shRNAs is reported in Figure 1.

**Figure 2:**
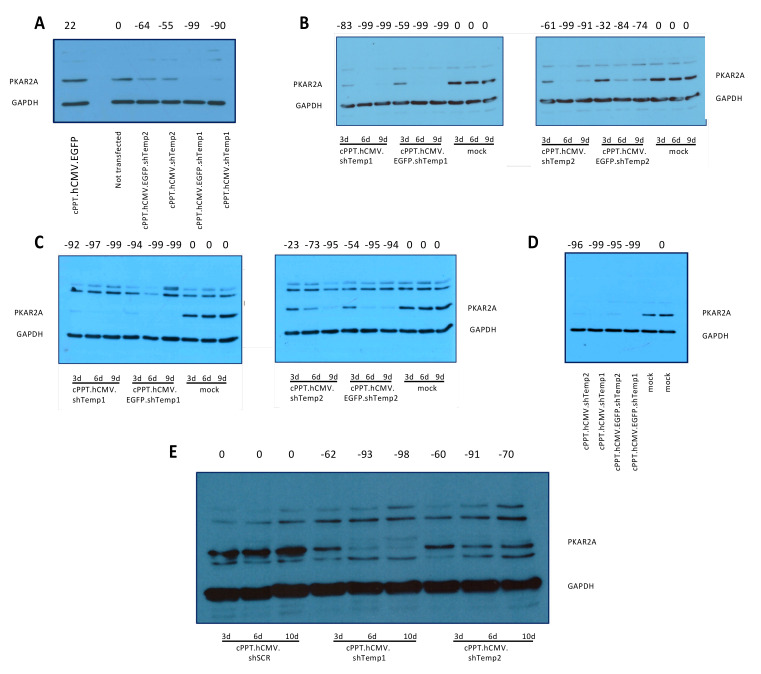
Developed lentiviral vectors mediate specific and long-lasting silencing of PKA. Western blot analysis of (**A**) 293T cells transfected with the developed lentiviral vectors analyzed 72 h posttransfection. (**B**–**E**) Western blot analysis of 293T (**B**) and U87 (**C**,**D**) cells, transduced with equivalent RT units of the recombinant lentiviral particles, as indicated, analyzed 3, 6, and 9 (**B**,**C**), 33 (**D**), or 3, 6, and 10 (**E**) days post-transduction. Bands were quantified and the percentage of reduction, calculated relative to non-transfected (**A**), mock (**B**–**D**) or scramble (shSCR, **E**), is indicated above each line. (**D**) the mean of two mock lanes was used. Primary antibodies: PKA R2A and GAPDH; secondary antibody: anti-rabbit HRP conjugated. Recombinant lentiviral particles lead to efficient silencing of PKA R2A in GBM cells.

Firstly, gene silencing efficiency of the recombinant lentiviral vectors carrying PKA R2A shRNA sequences was tested through transfection of 293T cells with the newly generated vectors. PKA R2A expression was evaluated 3 days post-transfection through Western blot analysis, showing an efficient R2A downregulation and a higher silencing efficiency of PKA R2A Temp1 compared to PKA R2A Temp2 shRNA sequence (Figure 2A).

Secondly, by calcium phosphate transfection of 293T cells, lentiviral particle stocks were produced for all recombinant lentiviral vectors carrying PKA R2A shRNA sequences. Vector stocks were quantified by RT assay and 293T cells were transduced with approximately 200,000 cpm RT units of recombinant lentiviral vectors in order to validate their PKA R2A gene silencing. Western blot analysis demonstrated an efficient PKA R2A downregulation up to 9 days post-transduction and confirmed a higher silencing efficiency of shTemp1 compared to shTemp2 sequence (Figure 2B).

Next, we moved to U87 human GBM cell line, one of the most extensively studied GBM cell models. Gene silencing capability of PKA R2A shRNA lentiviral vectors was assessed in U87 cells transduced with approximately 200,000 cpm of RT unit particles. An efficient and stable downregulation of PKA R2A expression was reported up to 30 days post-transduction, as well as a higher silencing efficiency of shTemp1 compared to shTemp2 sequence (Figure 2C,D).

In order to evaluate off-target effects induced by PKA R2A shRNAs, a scrambled shRNA (shSCR) was also designed and cloned within the same lentiviral vector described above. The shSCR vector stock was produced by calcium phosphate transfection of 293T cells and used in the subsequent experiments. 

Recombinant lentiviral particles (shSCR, shTemp1, shTemp2) were concentrated and 100,000 cpm RT units adopted to transduce U87 target cells. In these conditions, shTemp1 and shTemp2 transduction resulted in efficient and slight PKA R2A downregulation respectively, whereas shSCR vector transduction did not prove to significantly affect PKA R2A protein levels compared to mock transduced cells, as reported by Western blot analysis up to 10 days post-transduction (Figure 2E).

**Figure 3:**
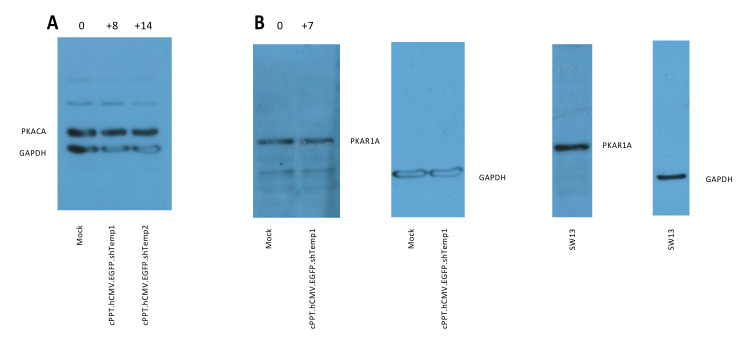
Western blot analysis of (**A**) PKA catalytic subunit in U87 cells 6 days after transduction; R2A silencing is shown in Supplementary Figure S1. (**B**) PKA R1A in U87 cells 33 days after transduction, same experiment as Figure 2D; SW13 cells are shown as a positive control for PKA R1A expression. The percentage of change relative to mock is shown above silenced lanes.

After transduction of U87 cells, no effect is apparent on PKA catalytic subunit (Figure 3A). Furthermore, PKA R1A subunit (Figure 3B) appears not affected by R2A silencing (see Figure 2D and Supplementary Figure S1).

**Figure 4:**
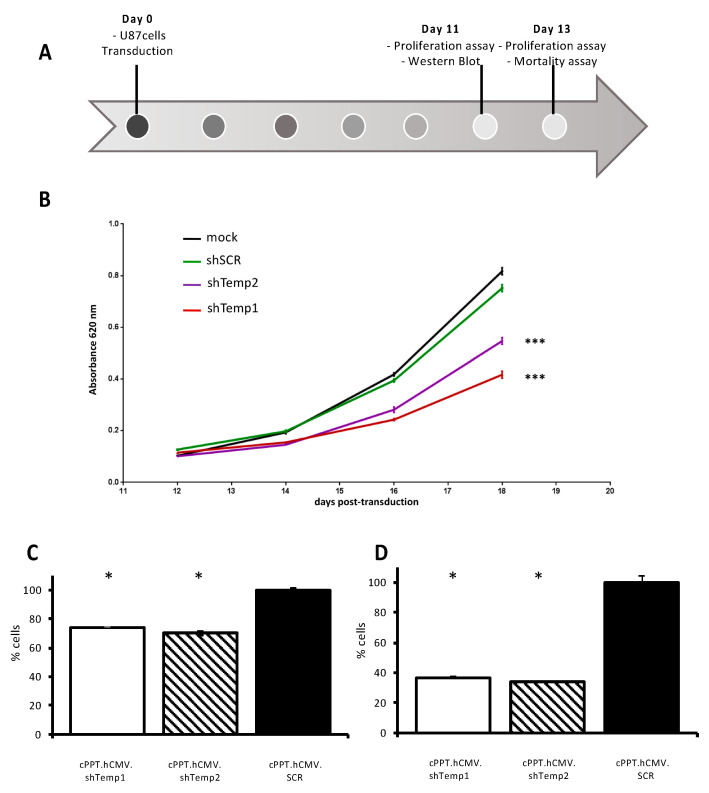
Effect of PKA silencing on U87 cells proliferation. (**A**) Timeline of transduction experiments in U87 cells. (**B**) MTT cell viability curves of U87 cells transduced with equivalent RT units of the lentiviral particles. On day 11, transduced U87 cells were processed for Western blot analysis of PKA R2A downregulation and seeded for MTT evaluation of cell growth. MTT assays were performed on day 12, 14, 16, and 18 post-transduction. Linear regression analysis of cell viability curves: shSCR versus shTemp1 and shTemp2: *** *p* < 0.001. (**C**,**D**) Analysis of proliferation of U87 cells 11 (**C**) and 13 (**D**) days after transduction. A significant reduction in cell number compared to scramble is present already 11 days post-transduction; * *p* < 0.05 versus scramble (black bar).

Next, functional effects of PKA R2A silencing were investigated in U87 human GBM non-stem cells (Figure 4A). Following Western blot confirmation of R2A downregulation in shTemp1- and shTemp2-expressing cells, MTT assays were performed to evaluate metabolic activity of U87 cells with silenced PKA R2A expression. MTT cell viability curves from 12 up to 18 days post-transduction indicated a significantly reduced cell viability of U87 cells with downregulated PKA R2A expression compared to cells transduced with the shSCR vector. More specifically, the more efficient was R2A silencing, the greater was U87 cell metabolism impairment, as reported for shTemp1 and shTemp2 transduced cells (Figure 4B). Conversely, no significant difference was detected between cells expressing the shSCR sequence and mock transduced cells, indicating that lentiviral transduction and shRNA expression did not significantly affect U87 cell viability. 

Proliferation was also analyzed in the same cell line and showed a significant reduction in proliferation with both shTemp1 and shTemp2 11 and 13 days after transduction (Figure 4C,D, respectively). However, mortality did not significantly increase, as indicated by FACS analysis of viable and apoptotic/necrotic cells (Supplementary Figure S2). This finding suggest that R2A silencing affects cell replication rate more than death.

**Figure 5:**
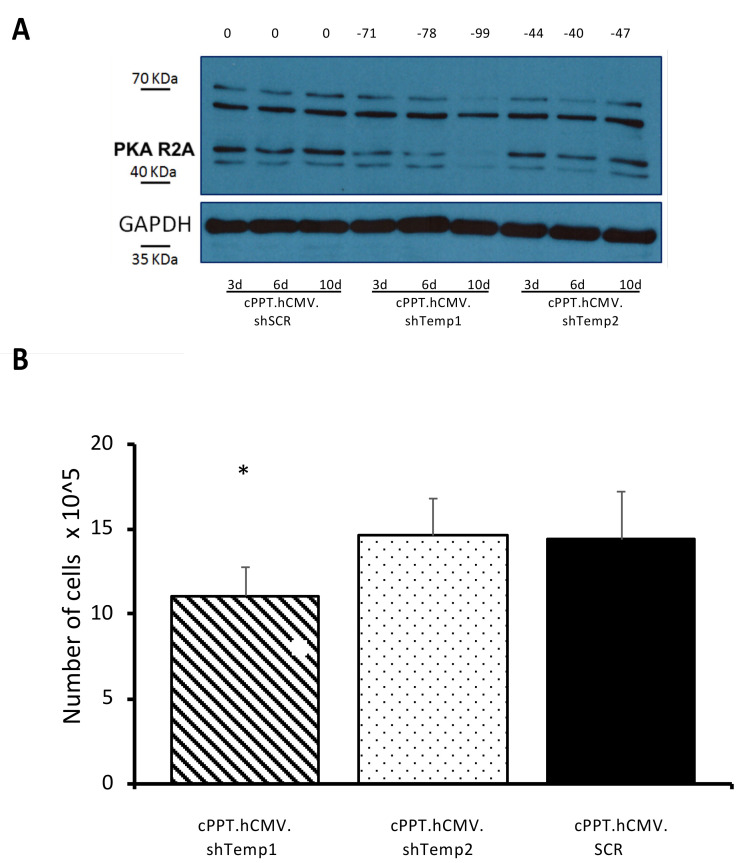
Recombinant lentiviral particles lead to efficient silencing of PKA R2A in GBM stem-like cells and affect their viability. (**A**) Western blot analysis of NCH421K cells transduced with equivalent RT units of recombinant lentiviral particles analyzed 3, 6 and 10 days post-transduction. Bands were quantified, the percentage of reduction is indicated above each lane and was calculated relative to scramble (shSCR) at the same time point. Primary antibodies: PKA R2A and GAPDH; secondary antibody: anti-rabbit HRP-conjugated. (**B**) Number of NCH421K viable cells 10 days post-transduction. Cells (1.0 × 10^5^) were transduced with equivalent RT units of the lentiviral particles. Viable cells raw number is reported, * *p* < 0.05.

The same optimized transduction conditions were then used to investigate PKA R2A silencing capability of our shRNA vectors in human GBM stem-like cells, representing nowadays a recognized model for studying many aspects of GBM biology [30,31]. PKA R2A downregulation was thus assessed in gliomaspheres of NCH421K human GBM stem-like cells, which present the same peculiar intracellular distribution of R2A subunit (data not shown) previously described in non-stem GBM cells [22,23]. A progressive reduction in PKA R2A protein levels was reported also for NCH421K cells transduced with shTemp1 vector, whereas no or minimal reduction in R2A expression was induced by shTemp2 up to 10 days post-transduction (Figure 5A).

Glioma stem-like NCH421K cells were analyzed 10 days post-transduction by trypan blue staining, followed by cell counting at the light microscope. Importantly, only shTemp1 expressing cells showed a significant reduction in the overall number of cells when compared to the shSCR transduced cells (Figure 5B) in the number of dead cells, in agreement with the PKA R2A silencing data in the same cell line (Figure 5A). 

Overall, these findings suggest a potential relationship between PKA R2A downregulation and cell proliferation in both a GBM cell line and in human GBM stem-like cells. 

**Figure 6:**
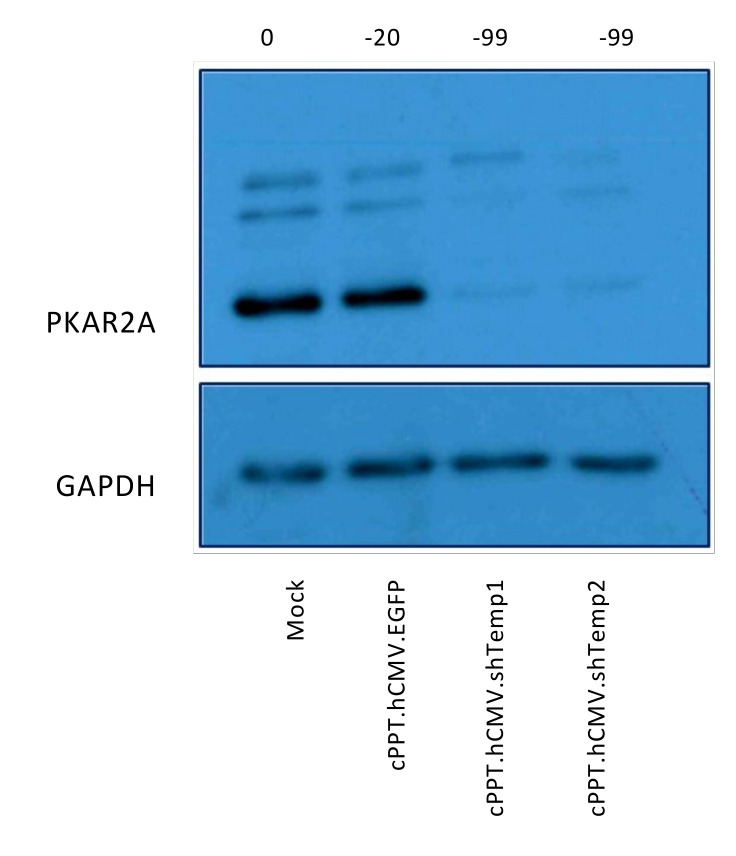
Recombinant lentiviral particles lead to efficient silencing of PKA R2A in primary GBM stem-like cells. Western blot analysis of primary cells transduced with equivalent RT units of recombinant lentiviral particles analyzed three months post-transduction. Bands were quantified, the percentage of reduction is indicated above each lane and was calculated relative to mock-infected cells. Primary antibodies: PKA R2A and GAPDH; secondary antibody: anti-rabbit HRP-conjugated.

A primary culture of glioblastoma stem-like cells was also analyzed: lentiviral vectors silenced PKA R2A efficiently, up to 3 months post-transduction (Figure 6).

Next, we interrogated the TCGA database, which reports data from 172 glioblastoma specimens, for possible correlations between the expression of PRKAR2A gene, which codes for R2A, and other selected genes. As it can be expected from the GBM-specific localization of PKA R2A in the Golgi apparatus, significant positive correlation was found between the expression of PRKAR2A and GOLGA1 and GOLGA4, but not for other Golgin genes. It correlated positively also with the PKA-docking AKAP12 protein, and the GBM-enriched NCAM1 (Supplementary Figure S3). However, no correlation was noted between PKAR2A and either VIM, which codes for vimentin, a stemness marker, or GFAP, while significant positive correlation was detected for the MKI67 proliferation marker and BCL2 anti-apoptotic gene (Supplementary Figure S4). Since it is possible that a downregulation of one PKA regulatory subunit leads to a compensatory increase in the expression of other regulatory subunits, we explored the expression of PRKAR2A relative to the one of the other 3 regulatory subunits (Supplementary Figure S5), finding no correlation with PRKAR1B and PRKAR2B, while a positive correlation was found with PRKAR1A. Of note, no correlation was observed between the expression of PRKAR2A and PRKACA, which codes for PKA catalytic alpha subunit.

## 4. Discussion

PKA tetramer consists of two regulatory subunits, responsible for docking at specific sites, and two catalytic subunits, responsible for phosphotransferase activity leading to PKA biological effects, and in glioblastoma the localization of PKA appears different from brain tissue [20]. Previous studies already pointed out the potential role of PKA R2A as a diagnostic marker for human GBM [22,23] and suggested the opportunity to reduce R2A expression in order to stimulate the expression of other PKA subunits, based on the assumption that the tumorigenic phenotype of GBM cells may be induced by PKA imbalance [22]. The data reported in the present study provide a proof of principle for such a therapeutic option, demonstrating a potential correlation between human GBM cell viability and PKA R2A protein levels. We also explored the correlation between the expression of PRKAR2A and other genes in glioblastoma tissue database: interestingly, PRKAR2A correlated with Golgin genes linked to vesicular membrane trafficking, and the R2-binding AKAP12. While expression of PRKAR2A is correlated with proliferation (MKI67) and anti-apoptotic genes (BCL2), it is not correlated with stemness-associated genes like VIM. It would be worthy of further investigation the lack of correlation between PRKAR2A and PKA catalytic or regulatory subunits beta isoforms, while the positive correlation between PRKAR2A and PRKAR1A suggests no compensatory increase between these two isoforms in untreated GBM tissue. It remains to be determined whether an artificially-induced downregulation of PKA R2A will affect the expression of the other regulatory isoforms. Consistently with the hypothesis of a therapeutic exploitation of the cAMP/PKA signaling pathway, Kang and colleagues also proposed the induction of growth arrest and forced neural differentiation via cAMP/PKA/CREB pathway in CD133-expressing GBM cancer stem cells as a new therapy for brain tumors [32]. Actually, multiple indications exist to support an increased efficacy of radiotherapy in synchronized GBM cells and animal models, and the quest for radiosensitizer strategies has led to different ongoing clinical trials [33]. In this framework, lentiviral vectors could be applied locally during surgery, as it happens now for carmustine wafers, to boost subsequent radiotherapy. Here, we demonstrate that our recombinant lentiviral vectors carrying PKA R2A shRNA sequences are able to efficiently downregulate R2A expression in human GBM non-stem as well as stem-like cells, while they do not apparently affect the expression of other PKA regulatory and catalytic subunits, similarly to what happens in human melanoma cells [34]. While more detailed studies are warranted, this rules out compensatory increase in the expression of other PKA subunits. Functionally, R2A silencing has an inhibitory effect on U87 and NCH421K cell growth. The possibility of targeting glioma stem cells appears intriguing for the possibility of long-term effects, given the high rate of recurrence of glioblastoma. Further studies will investigate whether R2A downregulation can also affect the proliferation of GBM cancer stem cells, as suggested by the correlation we found between R2A silencing and reduction in the number of cells. This aspect still deserves deeper investigation.

## 5. Conclusions

PKA is a conserved protein kinase, ubiquitously expressed in all mammalian cells and regulating a wide range of cellular functions. The well-known peculiarities of PKA R2A subunit in GBM cells may be exploited in a therapeutic setting for the design of novel targeted therapies. Our study describes an efficient lentiviral-mediated approach for stable downregulation of PKA R2A expression in GBM non-stem and stem-like cells and suggests a potential correlation between R2A silencing and a reduced viability of tumor cells. Therefore, our findings further support PKA R2A subunit as a promising target for anti-glioblastoma treatments to be combined with current conventional therapies.

## Data Availability

The data presented in this study are available in the article.

## Supplementary Materials

The following are available online at https://www.mdpi.com/article/10.3390/v13071361/s1, Figure S1: PKA R2A silencing in the same experiment shown in Figure 3 (main text). U87 cells, 6 days post-transduction. The percentage of reduction relative to mock is shown above each lane. Figure S2: annexin V-propidium iodide mortality assay 13 days after transduction: no significant change in mortality was detected in each condition. Figure S3: Correlation between PRKAR2A expression and the expression of genes GOLGA1 (A) and GOLGA4 (B) as Golgi markers, AKAP12 (C) as a PKAR2A docking protein, and NCAM1 (D) as a gene highly expressed in glioblastoma. Data on glioblastoma (*n* = 172) were retrieved from TCGA and analyzed with Xena browser. Significant positive correlations were found for GOLGA1, Pearson’s Rho = 0.2054, *p* = 0.0068, GOLGA4, Pearson’s Rho = 0.3178, *p* = 0.00002 and AKAP12, Pearson’s Rho = 0.2222, *p* = 0.0033, while a barely significant positive correlation was found for NCAM1, Pearson’s Rho = 0.1567, *p* = 0.0401. Figure S4: Correlation between PRKAR2A expression and the expression of genes VIM (A) as a stem cell marker, GFAP (B) as a marker of differentiated cells, MKI67 (C) as a proliferation marker, BCL2 (D) as an inhibitor of pro-apoptotic genes. Data on glioblastoma (*n* = 172) were retrieved from TCGA and analyzed with Xena browser. No significant correlation was found for VIM and GFAP, while significant positive correlations were found for MKI67, Pearson’s Rho = 0.2343, *p* = 0.0012, and BCL2, Pearson’s Rho = 0.1962, *p* = 0.0099. Figure S5 Correlation between PRKAR2A expression and the expression of genes PRKAR1A (A), PRKAR1B (B), PRKAR2B (C), PRKACA (D). Data on glioblastoma (*n* = 172) were retrieved from TCGA and analyzed with Xena browser. One slightly positive, yet significant correlation was found, between PRKAR2A and PRKAR1A: Pearson’s Rho = 0.1720, *p* = 0.024.
