# Supplementary material for: Targeting the Regulatory Subunit R2Alpha of Protein Kinase A in Human Glioblastoma through shRNA-Expressing Lentiviral Vectors
Source: Viruses. 2021 Jul 13;13(7):1361. doi: 10.3390/v13071361 (PMC8310305; doi:10.3390/v13071361)

Zorzan et al.

Targeting the regulatory subunit R2Alpha of protein kinase A in human glioblastoma through shRNA-expressing lentiviral vectors

Supplementary Figure S1: PKA R2A silencing in the same experiment shown in Fig. 3 (main text). U87 cells, 6 days post-transduction. The percentage of reduction relative to mock is shown above each lane.

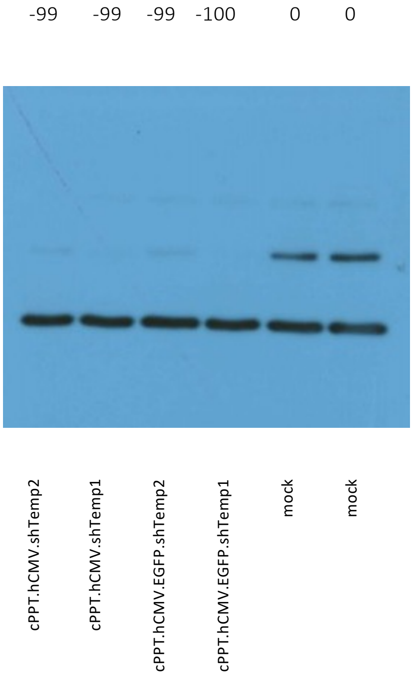

Supplementary Figure S2: annexin V-propidium iodide mortality assay 13 days after transduction: no significant change in mortality was detected in each condition.

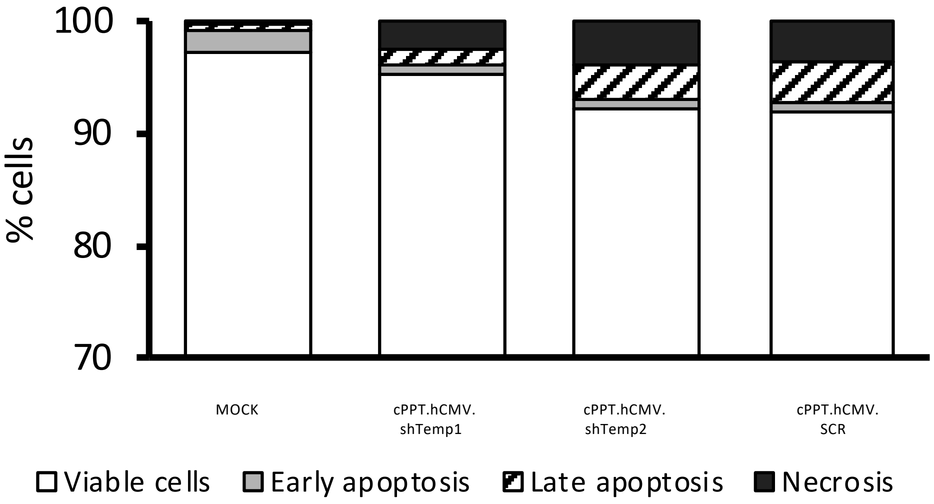

Supplementary Figure S3: Correlation between PRKAR2A expression and the expression of genes GOLGA1 (A) and GOLGA4 (B) as Golgi markers, AKAP12 (C) as a PKAR2A docking protein, and NCAM1 (D) as a gene highly expressed in glioblastoma.

Data on glioblastoma (n=172) were retrieved from TCGA and analyzed with Xena browser. Significant positive correlations were found for GOLGA1, Pearson's Rho=0.2054, p=0.0068, GOLGA4, Pearson's Rho=0.3178, p=0.00002 and AKAP12, Pearson's Rho=0.2222, p=0.0033, while a barely significant positive correlation was found for NCAM1, Pearson's Rho=0.1567, p=0.0401.

A

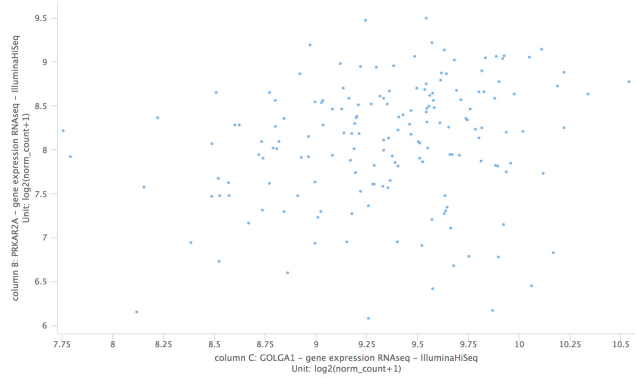

B

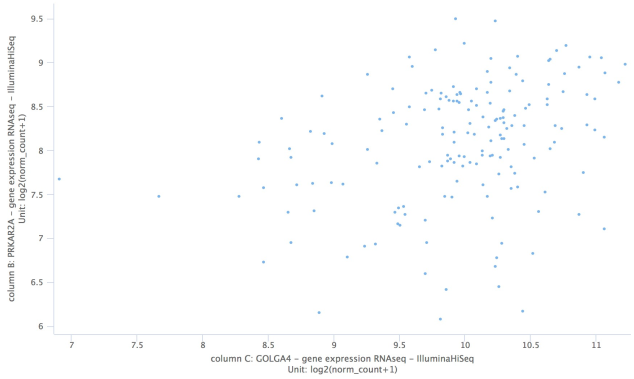

C

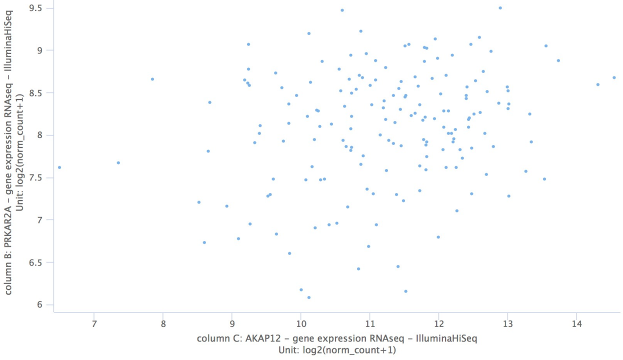

D

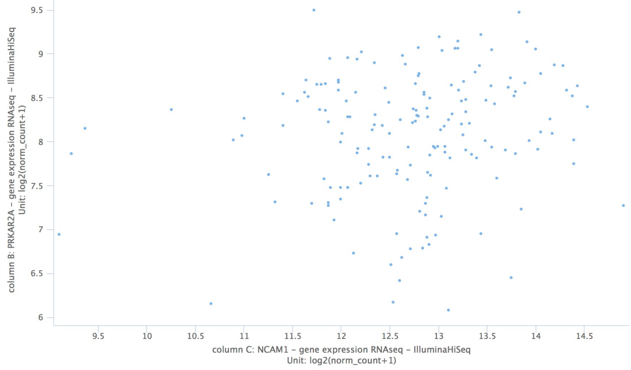

Supplementary Figure S4: Correlation between PRKAR2A expression and the expression of genes VIM (A) as a stem cell marker, GFAP (B) as a marker of differentiated cells, MKI67 (C) as a proliferation marker, BCL2 (D) as an inhibitor of pro-apoptotic genes.

Data on glioblastoma (n=172) were retrieved from TCGA and analyzed with Xena browser. No significant correlation was found for VIM and GFAP, while significant positive correlations were found for MKI67, Pearson's Rho=0.2343, p=0.0012, and BCL2, Pearson's Rho=0.1962, p=0.0099.

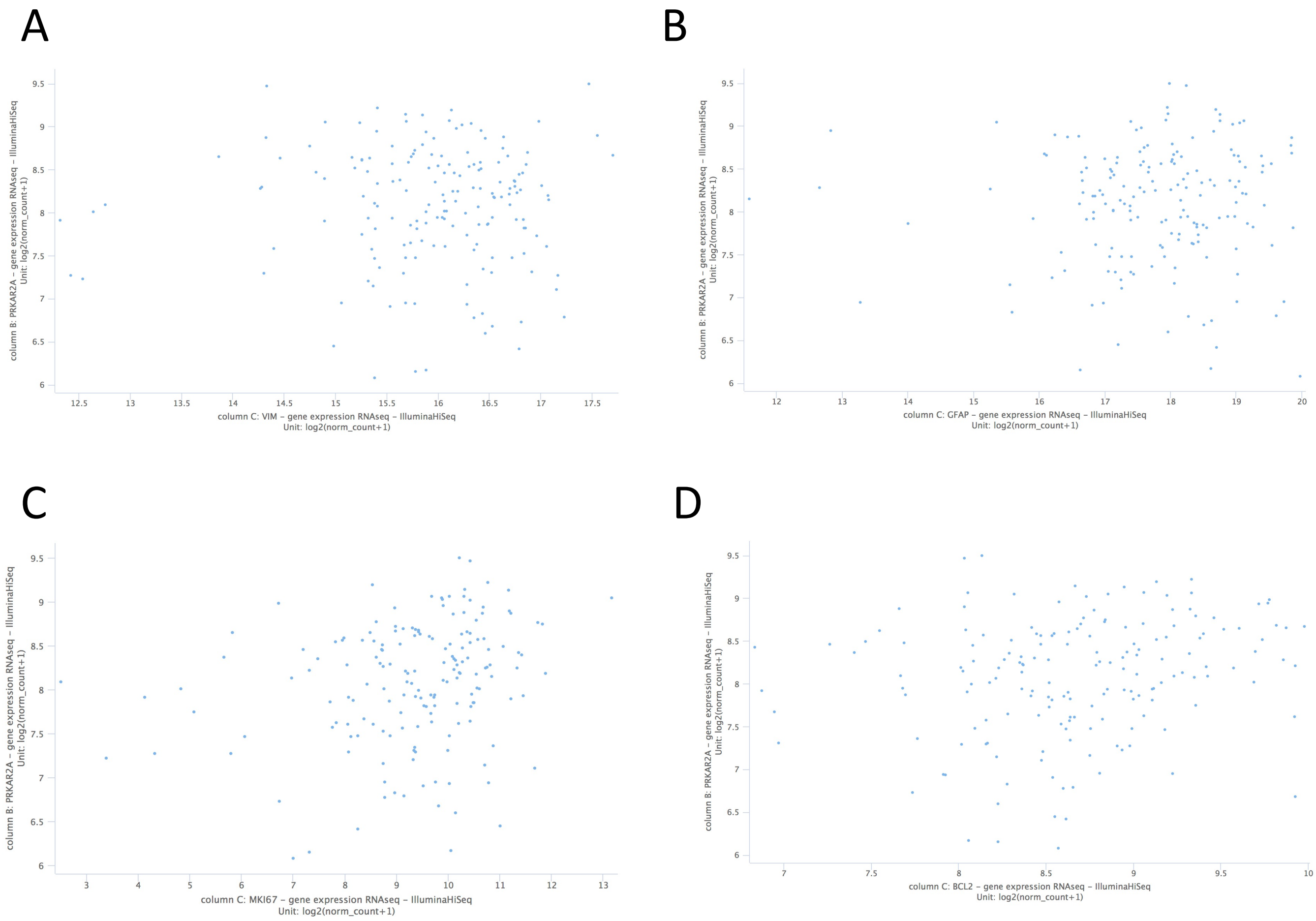

Supplementary Figure S5: Correlation between PRKAR2A expression and the expression of genes PRKAR1A (A), PRKAR1B (B), PRKAR2B (C), PRKACA (D).

Data on glioblastoma (n=172) were retrieved from TCGA and analyzed with Xena browser. One slightly positive, yet significant correlation was found, between PRKAR2A and PRKAR1A: Pearson's Rho=0.1720, p=0.024.

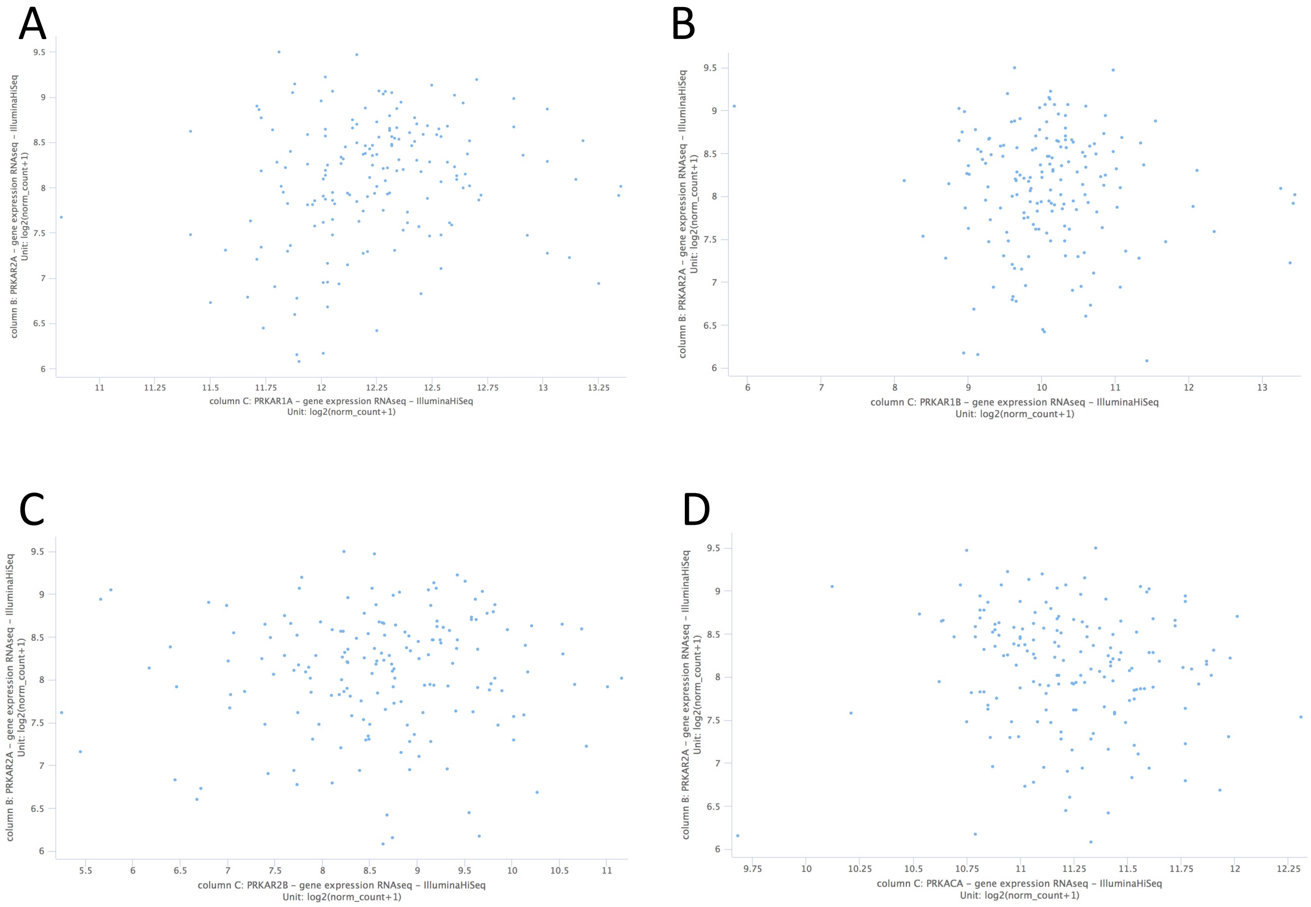

ORIGINAL BLOT FIGURE 2

A

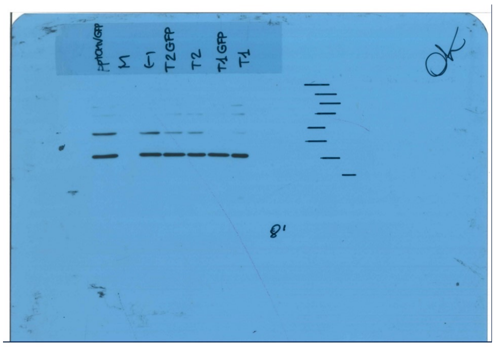

B

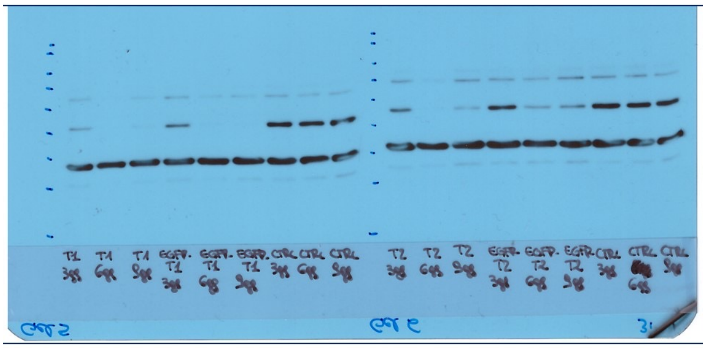

C

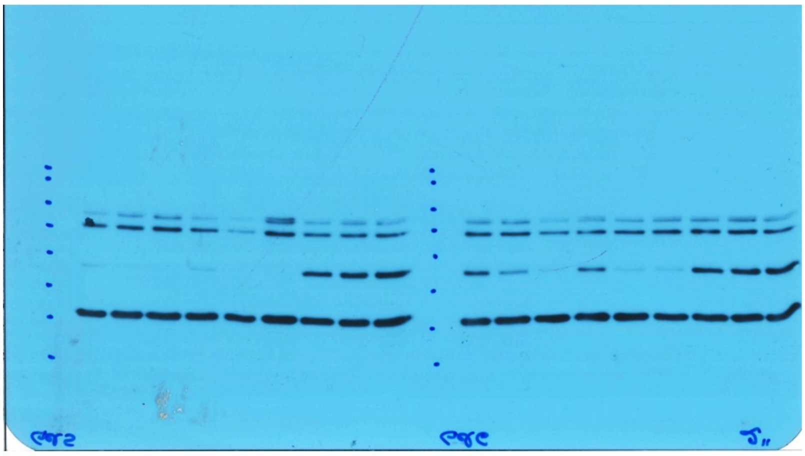

D

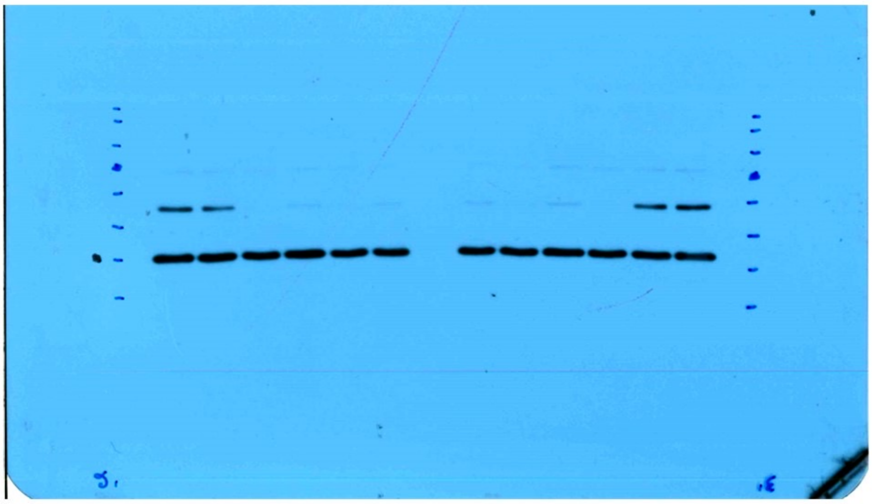

E

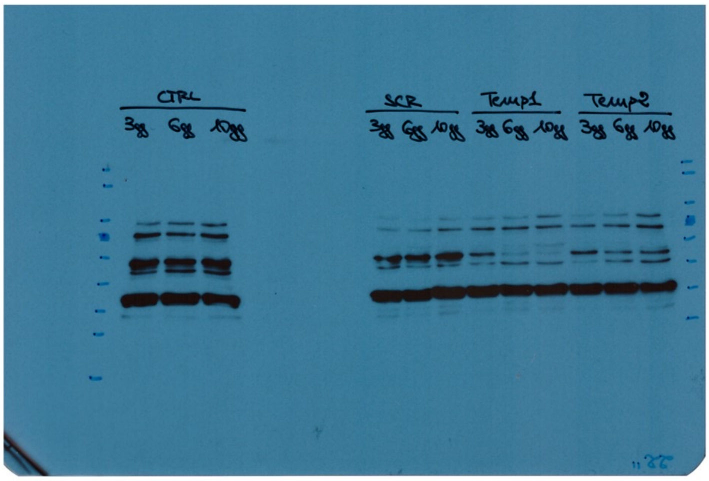

ORIGINAL BLOT FIGURE 3

A

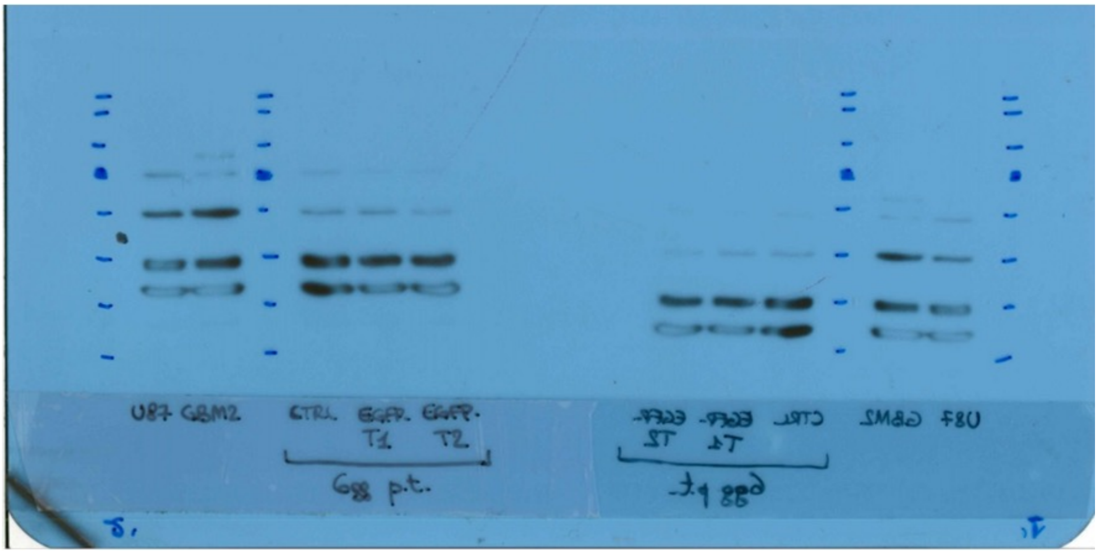

B

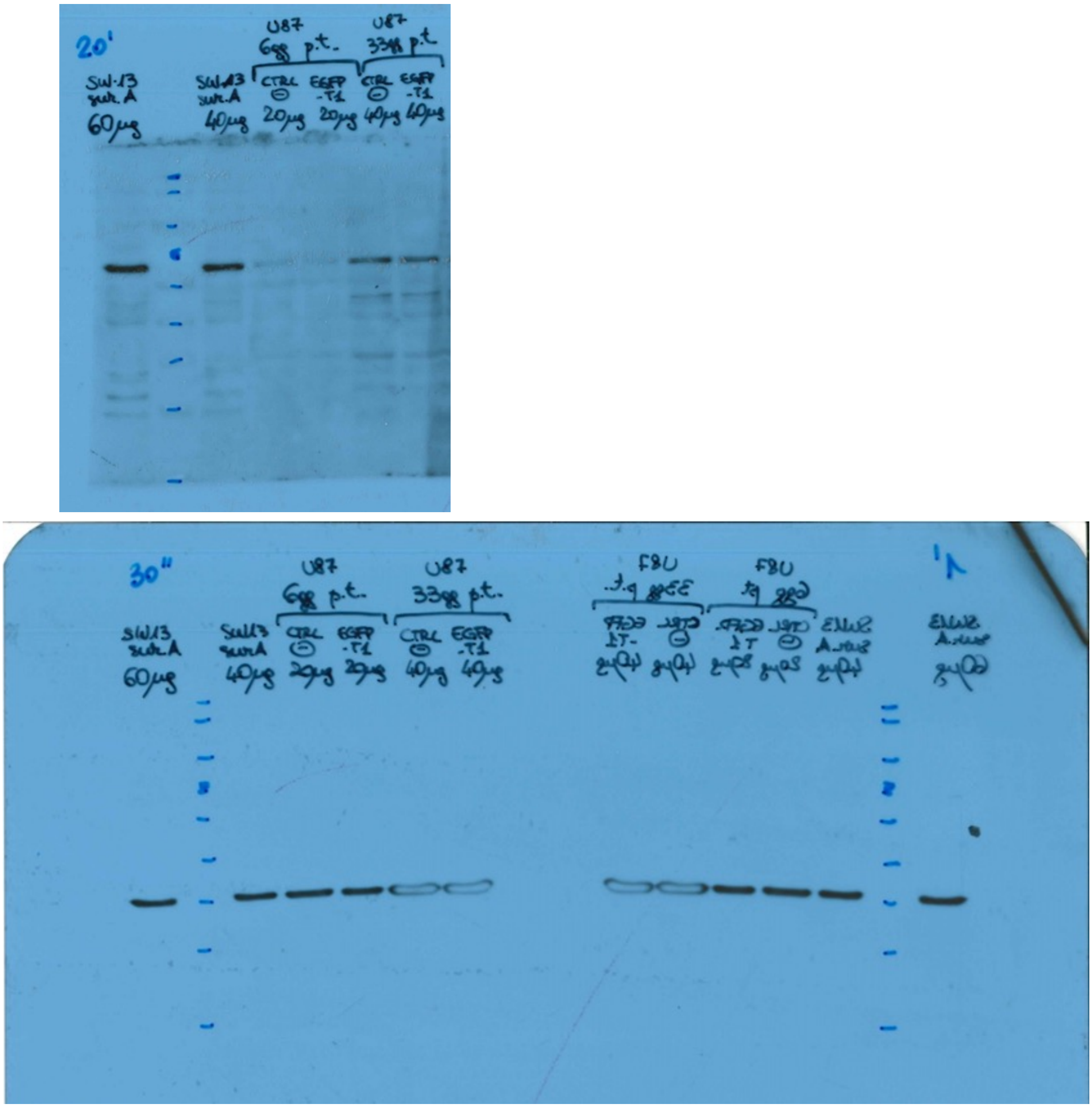

ORIGINAL BLOT FIG. 5

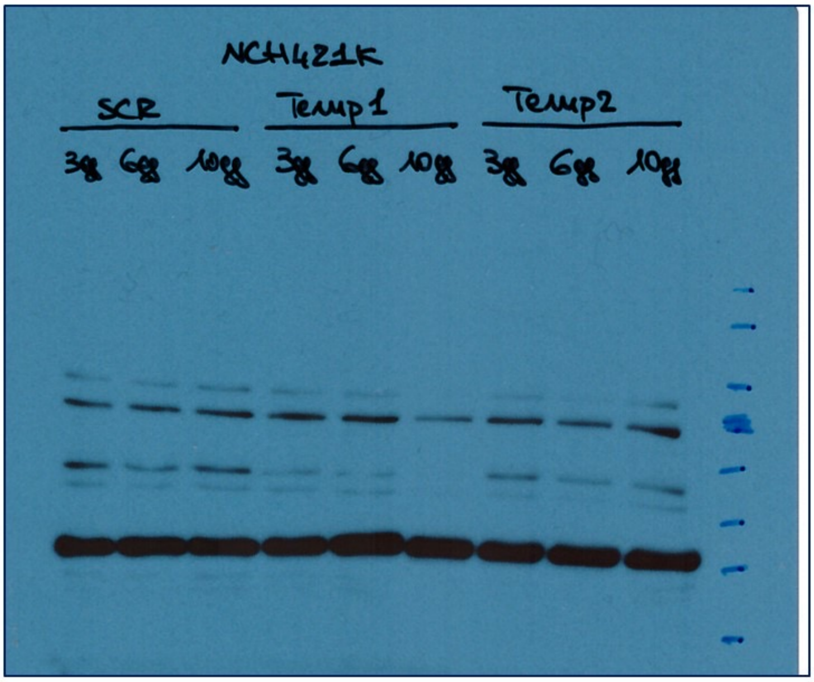

ORIGINAL BLOT FIG. 6

PKA R2A

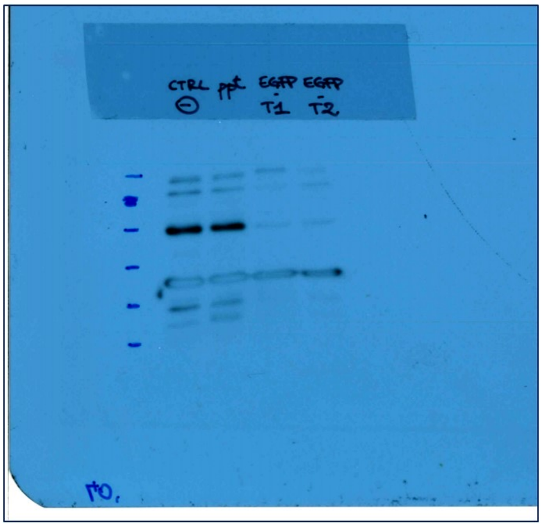

GAPDH

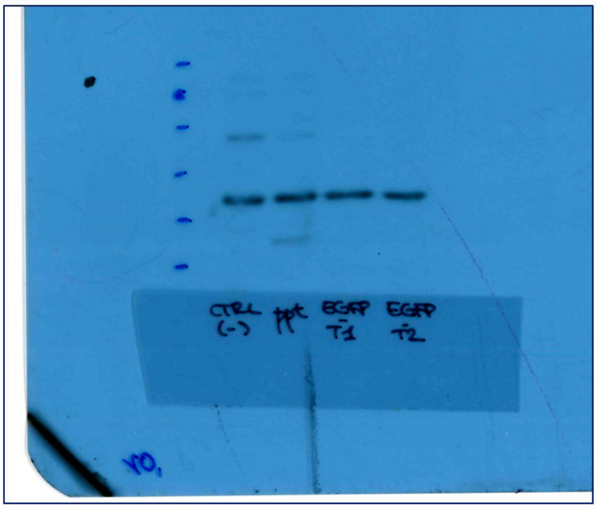

Supplement: Supplementary file 1 [file viruses-13-01361-s001.zip › viruses-1181267-supplementary.pdf]
